# Plasma Sclerostin Levels in Rheumatoid Arthritis Women on TNF-α Inhibitor Therapy

**DOI:** 10.3390/ph17060666

**Published:** 2024-05-22

**Authors:** Anna Szeremeta, Agnieszka Jura-Półtorak, Aleksandra Zoń-Giebel, Krystyna Olczyk, Katarzyna Komosińska-Vassev

**Affiliations:** 1 Department of Clinical Chemistry and Laboratory Diagnostics, Faculty of Pharmaceutical Sciences in Sosnowiec, Medical University of Silesia, Katowice, Jedności 8, 41-200 Sosnowiec, Poland; ajura@sum.edu.pl (A.J.-P.); olczyk@sum.edu.pl (K.O.); kvassev@sum.edu.pl (K.K.-V.); 2 Department of Rheumatology and Rehabilitation, Specialty Hospital No. 1, Żeromskiego 7, 41-902 Bytom, Poland; azongiebel@gmail.com

**Keywords:** sclerostin, bone loss, rheumatoid arthritis, biological therapy, TNF-α inhibitors

## Abstract

Rheumatoid arthritis (RA) is associated with significant systemic and local bone loss. The aim of this study was to assess whether or not 15-month tumor necrosis factor α inhibitor (TNFαI) therapy in combination with methotrexate (MTX) affects circulating levels of sclerostin (SOST) in female RA patients. Plasma levels of SOST were measured using immunoassays kits. Baseline SOST levels showed no significant differences between RA patients and control participants. Postmenopausal women with RA tended to have higher sclerostin levels than premenopausal woman with RA. After 15 months of treatment with TNFαI, plasma levels of SOST were decreased. Before starting biological therapy, circulating levels of SOST significantly correlated with the patient’s age (*p* < 0.05) and the marker of inflammation, such as ESR (*p* < 0.05). Multivariate regression analysis showed that age was the only significant predictor for baseline SOST levels in women with RA (β = 0.008, *p* = 0.028, R2 model = 0.293). Moreover, a positive correlation between SOST levels and the 28 joint disease activity score value based on the erythrocyte sedimentation rate (DAS28-ESR) was found at baseline (*p* < 0.05), as well as after 15 months of biological therapy (*p* < 0.05). Thus, plasma SOST levels may be helpful for monitoring the efficacy of TNFαI treatment in RA patients. According to our results, TNFαI, in combination with MTX, has a beneficial effect on bone turnover with a significant reduction in bone metabolism marker SOST.

## 1. Introduction

Rheumatoid arthritis (RA) is a polyarticular chronic inflammatory disorder that affects about 1% of the world’s population, and occurs two-to-three-fold more frequently in women than in men [1]. While most types of arthritis other than rheumatoid arthritis (e.g., spondyloarthritis, psoriatic arthritis or even osteoarthritis) share a common tendency to induce significant alterations in cartilage remodeling, a central feature of RA is the presence of periarticular bone erosion, which results from excessive local bone resorption and inadequate bone formation [2,3]. Tumor necrosis factor α (TNF-α) is one of the main proinflammatory cytokines that plays a crucial role in the inflammatory response and systemic and localized bone loss in RA by targeting the Wingless (Wnt) signaling pathway [2,3,4]. Notably, dysregulation of the Wnt signaling pathway has been described to be implicated in reduced osteoblast function and suppressed bone repair in both human RA and murine models of arthritis [3,4,5]. Wnt proteins play an important role in bone development and metabolism via two signaling pathways: the β-catenin-dependent canonical pathway and β-catenin-independent noncanonical pathway. Canonical Wnt signaling is involved in the regulation of osteoblast proliferation, differentiation and survival. Importantly, activation of the Wnt/β-catenin pathway in osteoblasts suppresses osteoclastogenesis through the down-regulation of the receptor activator of nuclear factor κΒ ligand (RANKL)/osteoprotegerin (OPG) ratio, thereby inhibiting bone resorption [4,5,6,7].

**Figure 1:**
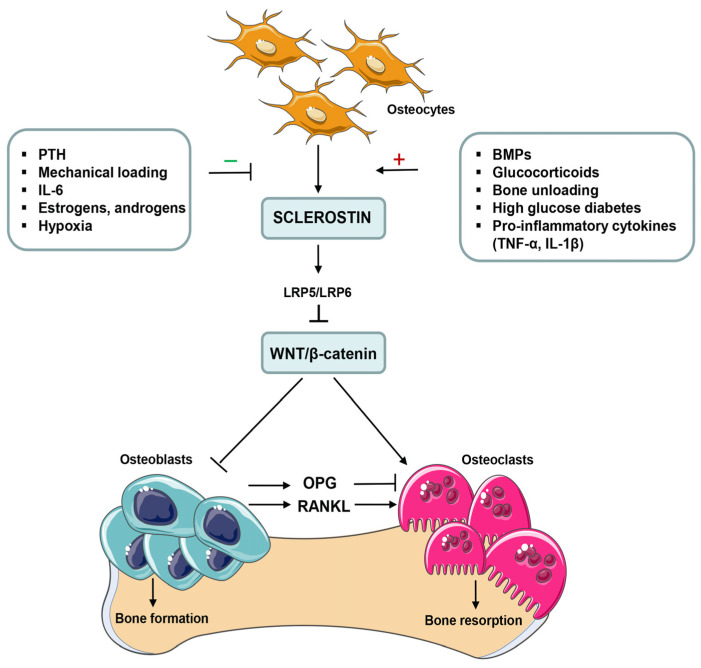
Positive and negative regulators of sclerostin (SOST) expression and its effects on bone cells. The expression of sclerostin is positively and negatively regulated by various local and systemic factors as well as mechanical stimulation on bone tissue. Sclerostin, encoded by the SOST gene, is primarily secreted by mature osteocytes during the bone remodeling process. It binds to low-density lipoprotein receptor-related protein 5/6 (LPR5/6), and affects the interaction between Wnt ligands and LRP5/6, which inhibits Wnt/β-catenin signals and inhibits osteoblast differentiation and proliferation. Sclerostin also regulates bone resorption through the suppression of osteoprotegerin (OPG) expression and the induction of receptor activator of nuclear factor κB ligand (RANKL) expression in osteoblasts. In addition, inhibition of Wnt/β-catenin pathway in osteoclast precursors directly promotes their differentiation and enhances osteoclastogenesis. BMPs, bone morphogenetic proteins; IL, interleukin; PTH, parathyroid hormone; TNF-α, tumor necrosis factor α.

Numerous animal and human studies suggest that the expression of sclerostin (SOST), one the most potent inhibitors of the Wnt pathway, may affect bone formation and bone mass in normal and pathological stages. Overexpression of sclerostin in genetically manipulated mice causes a remarkable reduction in bone mass and strength. In contrast, a deficiency of sclerostin in two rare genetic bone diseases, such as sclerosteosis and van Buchem’s disease, is associated with a high-bone-mass phenotype and a low risk of fractures [4,5,7]. Sclerostin, encoded by the SOST gene, is primarily secreted by mature osteocytes and some chondrocytes [4,5,6,7]. However, it has also been proposed that fibroblast-like synoviocytes (FLSs) may be the main source of circulating sclerostin in patients with RA [8]. This protein is upregulated by proinflammatory cytokines during inflammation, interacts with the extracellular domains on low-density lipoprotein receptor-related protein 5/6 (LRP5/6), and displaces canonical Wnt signaling proteins, thereby reducing osteoblastic bone formation [5,6,7]. Regulation of sclerostin expression and its role in bone remodeling are shown in Figure 1.

Several clinical studies have shown that serum sclerostin levels increase with age and after menopause, potentially contributing to increased fracture risk in postmenopausal women [9,10,11]. Based on these findings, it can be assumed that the inflammatory background of rheumatoid arthritis may contribute to the upregulation of sclerostin, resulting in increased joint damage and bone erosion in RA patients. However, the literature reveals contradictory results regarding the relationship between sclerostin expression and bone loss in animal models of RA [4,5,7]. Likewise conflicting findings exist concerning sclerostin serum concentrations in RA patients [12].

Biological therapy based on TNF-α blockade is known to retard, or even arrest, bone erosion and inhibit radiographic progression in RA patients [3,4]. Nevertheless, the mechanism by which TNFαI effectively reduces structural damage is not fully understood. Furthermore, data concerning the effects of TNFαI on plasma levels of sclerostin, a physiological inhibitor of bone formation in RA patients, still remain inconsistent. Therefore, the aim of this study was to assess whether or not long-term anti-TNF-α therapy in combination with methotrexate (MTX) affects circulating levels of sclerostin in female RA patients.

## 2. Results

### 2.1. Clinical Response to TNFαI Therapy

**Figure 2:**
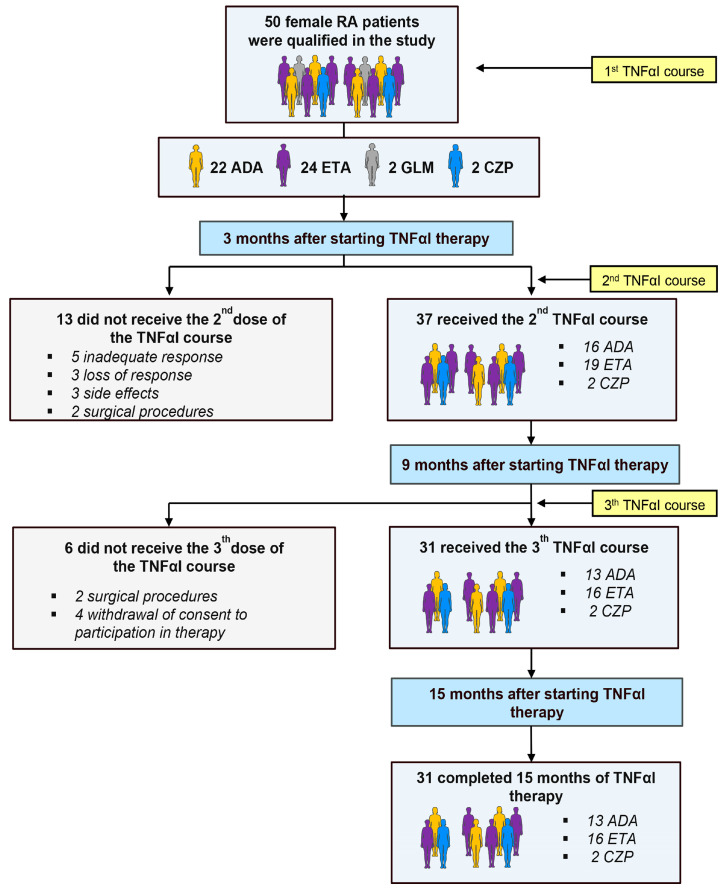
Flow chart of patients with rheumatoid arthritis (RA) treated with TNFαI, and reasons for dropout. ADA, adalimumab; ETA, etanercept; CZP, certolizumab pegol; GLM, golimumab; RA, rheumatoid arthritis; TNFαI, tumor necrosis factor α inhibitors.

Table 1 depicts the demographic and clinical characteristics, as well as the laboratory findings, of RA patients who completed the 15 months of anti-TNF-α treatment, who were obtained from our previous investigation [13]. Out of a total 50 women with RA, 19 (38%) participants discontinued the use of TNFαI and were thus excluded from this analysis; the remaining 31 (62%) women completed the 15-month follow-up period and were included in this study (Figure 2).

During the 15-month TNF-α-blocking therapy, a substantial clinical improvement in all female patients with RA (*n* = 31) was observed. TNFαI therapy resulted in significant reductions in clinical parameters, such as the disease activity score (DAS) involving 28 joints and, using the erythrocyte sedimentation rate (DAS28-ESR), the tender joint count (TJC), swollen joint count (SJC), and visual analogue scale (VAS) after 15 months of treatment compared with the baseline (Table 2). Furthermore, significant reductions in C-reactive protein (CRP) and ESR were noticed in all RA participants following the biological therapy (Table 2). Almost 84% (*n* = 26) of patients achieved complete remission (DAS28 value ≤ 2.6) and 16% (*n* = 5) saw at least low disease activity (DAS28 value < 3.2) at the 15th month of treatment (Table 2).

### 2.2. Effects of TNFαI Therapy on Circulating Sclerostin Levels

**Figure 3:**
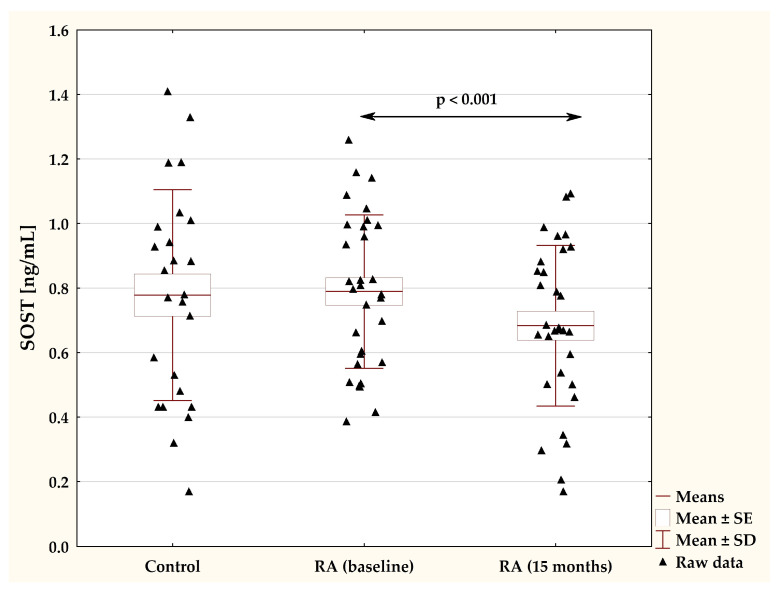
Plasma levels of SOST in RA patients before the start of TNFαI treatment and at the 15 month of treatment, and in control subjects. RA, rheumatoid arthritis; SOST, sclerostin; TNF-α, tumor necrosis factor α.

The plasma concentrations of sclerostin in female patients with RA at the baseline and during 15-month TNFαI treatment and in the control group are presented in Figure 3. Before the start of biological treatment, no significant difference was observed in serum SOST levels between RA women and the healthy participants (*p* = 0.883; Figure 3). Long-term administration of TNFαI resulted in a significant reduction in sclerostin concentrations compared with the baseline values (0.79 ± 0.24 vs. 0.68 ± 0.25 ng/mL, *p* < 0.001; Figure 3). Moreover, at 15 months, serum sclerostin levels were still not different from those in healthy subjects (*p* = 0.222; Figure 3).

**Figure 4:**
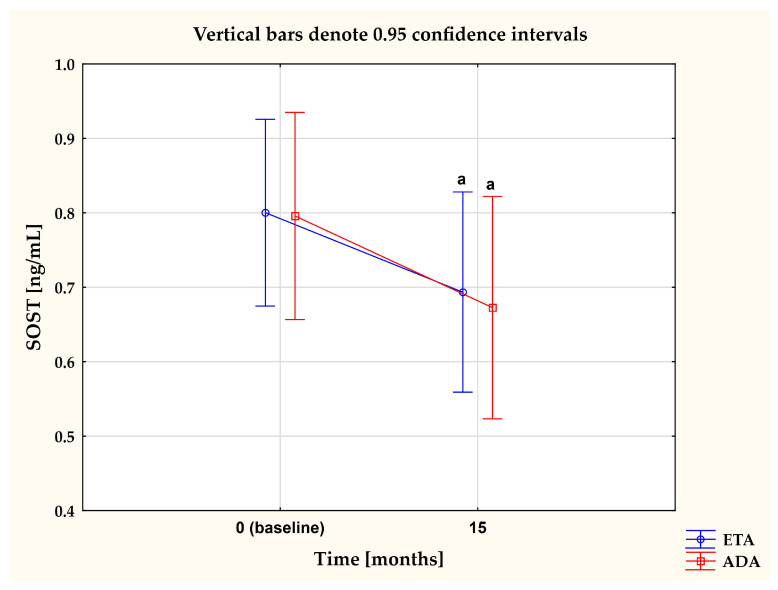
The impact of 15-month treatment with ETA and ADA on plasma SOST levels in RA patients. ^a^ *p* < 0.05. ADA, adalimumab; ETA, etanercept; RA, rheumatoid arthritis; SOST, sclerostin.

To evaluate whether or not the type of anti-TNF-α drug affected the levels of one the most potent inhibitors of the Wnt pathway, sclerostin, we compared changes in plasma levels of SOST in women with RA who continued with the first inhibitor (etanercept (ETA) or adalimumab (ADA)) for 15 months. Results are reported in Figure 4. Overall, both TNFαI drugs led to a remarkable decrease in plasma concentrations of SOST (both *p* < 0.05; Figure 4). Meanwhile, there were no significant changes in SOST levels between the ETA and ADA groups (*p* = 0.784; Figure 4).

### 2.3. Plasma Sclerostin Levels in Pre- and Postmenopausal Women with RA

When RA patients were analyzed in terms of the stage of menopause, plasma sclerostin levels in postmenopausal women with RA exhibited a tendency to increase both before (0.88 ± 0.22 ng/mL) and after 15 months of anti-TNF-α therapy (0.78 ± 0.20 ng/mL) compared with those in premenopausal RA women (0.72 ± 0.24 ng/mL and 0.61 ± 0.26 ng/mL, respectively; both *p* = 0.06; Table 3). Furthermore, sclerostin levels significantly decreased in response to the TNFαI treatment in both pre- and postmenopausal women with RA (both *p* < 0.05, Table 3).

### 2.4. Correlations between Sclerostin and Demographic, Clinical and Laboratory Parameters in RA Patients under TNFαI Treatment

The correlations among sclerostin and the other studied parameters in RA patients are presented in Table 4.

Sclerostin was significantly correlated with patients’ age (r = 0.490; *p* < 0.05) and tended to correlate with disease duration (r = 0.342; *p* = 0.059) (Table 4). Moreover, baseline sclerostin levels were significantly and positively correlated to the reduction in DAS28-ESR (r = 0.417; *p* < 0.05) and ESR (r = 0.428; *p* < 0.05), respectively (Table 4). After 15 months of biological treatment, a correlation between SOST levels and DAS28-ESR was still significant (r = 0.468; *p* < 0.05; Table 4), but not with ESR (r = 0.220; *p* = 0.235; Table 4). No significant associations between serum sclerostin levels and other monitored variables were found.

Furthermore, multiple regression analysis was performed to investigate whether or not age, ESR and DAS28-ESR were associated with baseline levels of sclerostin in RA patients (Table 5). The output of analysis showed the significant association of baseline sclerostin with RA patients’ age (β = 0.008, *p* = 0.028, and R2 model = 0.293).

## 3. Discussion

Since it has been shown that sclerostin is expressed under inflammatory conditions, we expected higher levels of sclerostin in women with RA, as a positive correlation has been established between inflammation and sclerostin expression by osteocytes [14,15]. In clinical practice, this relationship has also been observed in ankylosing spondylitis and in adult patients with juvenile idiopathic arthritis [16]. However, studies investigating the association of sclerostin with RA revealed controversial results. For example, serum concentrations of sclerostin in patients with RA have previously been reported as high [17,18,19,20] or normal [21,22,23,24,25]. In the present study, no significant difference was found in sclerostin levels between female RA patients and gender-matched healthy controls. These results are consistent with those of previous studies by Świerkot et al. [22] and Vargas-Munoz et al. [23], who also reported no difference in sclerostin levels between female RA patients and healthy subjects. Additionally, in a study conducted by Mehaney et al. [21] among 40 Egyptian RA patients (70% female and 30% male) with an average age of 48.9 ± 11.6 years, they found no significant difference in serum sclerostin levels between RA patients and controls. Similarly, Lim et al. [24] showed that sclerostin levels were not different in RA patients treated chronically with synthetic disease-modifying antirheumatic drugs (DMARDs) and glucocorticoids prior to etanercept therapy compared with those in healthy controls. Contrarily, the study by Dhakad et al. [26] demonstrated higher serum levels of sclerostin in female RA patients than in healthy controls. Researchers in other studies also reported increased serum sclerostin levels in patients with RA [17,18,19]. Such major discrepancies in SOST levels found by the different authors may be explained by various physiological and pathological factors that can affect circulating levels of this marker. First, advancing age is important among these factors because it induces a progressive increase in serum levels of SOST [9,11]. Second, as an estrogen signaling pathway negatively regulates SOST expression [10], age-related estrogen deficiency may contribute to an increase in circulating sclerostin levels in elderly women. Several clinical studies reported a significant increase in serum sclerostin levels with advancing age and after menopause, suggesting that serum SOST may be associated with aging- and estrogen deficiency-induced bone loss [9,10,11]. These findings are consistent with our results since we observed a significant correlation between sclerostin levels and the age of female RA patients. Furthermore, in the present study, postmenopausal women with RA displayed a tendency to have higher sclerostin levels both before and after TNFαI therapy compared with premenopausal woman with RA. This inclination towards higher plasma sclerostin levels in postmenopausal women may be a cause or/and a consequence of the increasing bone turnover and bone loss characteristic of the postmenopausal state. It is well known that estrogen deficiency following ovariectomy (OVX) amplifies T cell activation, leading to increased TNF-α levels in the bone microenvironment, thereby indirectly enhancing osteoclastogenesis [27]. Additionally, the progression of inflammatory symptoms in RA is accompanied by an increase in the production of proinflammatory cytokines, consequently upregulating sclerostin expression, which inhibits the repair of bone erosion by suppressing bone formation [2,4]. In light of these findings, our study showed that circulating sclerostin levels were significantly correlated with markers of RA activity, such as DAS28-ESR and ESR. This is comparable to a previous study by Gharbia et al. [18], who reported that serum sclerostin levels positively correlated with TJC, SJC, ESR, CRP and DAS28 in patients with RA. Ibrahim et al. [28] also showed a significant positive correlation between serum sclerostin levels and ESR, CRP and DAS28 in RA patients. Similar observations were made by Brabnikova-Maresova et al. [16] in adult patients with juvenile idiopathic arthritis. However, they did not detect any significant correlation between serum sclerostin and markers of inflammation [16]. On the other hand, several reports demonstrated that serum sclerostin levels were inversely correlated with DAS28 and/or CRP [24,29]. Moreover, there are studies that have reported no relationship between sclerostin levels and markers of RA activity [17,19,21,26]. These variable results could be due to differences in the disease activity of RA patients, disease durations and the type of anti-rheumatic drugs used.

Third, as sclerostin expression is strictly confined to osteocytes, it is possible to speculate that the lack of increased levels of sclerostin in RA patients might stem from excessive osteocyte death. Notably, Appel et al. [30] demonstrated that RA is marked by increased osteocyte death, as reflected by a high number of empty osteocytic lacunae in bone specimens from patients with RA. Therefore, the ability of osteocytes to synthesize and release sclerostin into the circulation of RA patients could be reduced. Finally, it should be noted that the differences in commercially available assays used to measure circulating sclerostin may partly explain the variability in findings [31,32].

Regarding the effects of TNFαI blockade on the circulating levels of sclerostin in RA patients, our data are consistent with those from several previous studies. Fassio et al. [33] demonstrated that the effective therapy of RA with certolizumab pegol (CZP) plus MTX significantly reduced serum levels of sclerostin and dicckopf-1 (DKK-1) in RA patients. These changes were related to the beneficial effect of TNFα inhibition with CZP on the levels of bone turnover biomarkers, which indicates an important mechanism for preventing systemic bone loss in RA patients [33]. Similar results were reported in study conducted by Cauli et al. [20], who described significantly lower serum SOST levels in patients with active RA at 24 weeks after initial adalimumab administration. Brabnikova-Maresova et al. [16] also observed this positive effect in the serum of young adult patients with JIA after 1-year and 2-year treatment with infliximab, ETA or ADA. However, other researchers did not find any effect of 6-month anti-inflammatory therapy with TNFαI [34,35]. Finally, Lim et al. [24] and Gulyás et al. [36] reported an increase in serum SOST levels in RA patients after 12 weeks and 12 months of TNFαI treatment, respectively. As mentioned earlier, the possible explanation for these contrasting results may lie in the methodical differences and the heterogeneity of the populations included, as well as the types of DMARDs used.

As sclerostin is a negative regulator of bone growth, the decreased levels of sclerostin following effective anti-TNF-α therapy seem to play a significant role in preventing systemic bone loss in RA patients. Indeed, in our earlier investigation [13], we reported an increase in the N-terminal propeptides of type I procollagen (PINP)/C-terminal crosslinking telopeptides of type I collagen (CTX-I) ratios in female RA patients after 15 months of TNFαI treatment compared with the baseline, suggesting an increase in osteoblast activity and a return to the balanced coupling of bone resorption and bone formation. However, we found no significant difference in the bone mineral density of vertebrae (L2-L4) and the femoral neck, which may indicate effective anti-TNFα therapy in women with RA stabilized bone loss within 15 months [13]. It can be assumed that this beneficial effect may be to with the ability of TNFαI to promote programmed cell death. As reported previously, etanercept therapy induces apoptosis of FLSs, which are likely a constant source of sclerostin in RA patients [37]. In addition, TNF-α blockade has been shown to suppress DKK-1 expression, further inhibiting SOST production [36]. Moreover, a positive association between serum sclerostin levels and markers of disease activity suggests that aggressive control of inflammation may begin to restore homeostasis in bone metabolism in patients with RA.

Our study has some limitations to consider. First of all, the sample size was relatively small, which may have reduced the power of this study. Moreover, the effect of CZP on sclerostin levels was not assessed due to the limited number of RA patients treated with this drug who completed 15 months of biological therapy, which amounted to only two individuals. Although 31 participants exhibited a positive response to anti-TNF therapy, the group was heterogeneous, consisting of pre- and postmenopausal female patients with RA. Third, the study’s design lacked a group of female RA patients who did not respond to TNFαI therapy, precluding a comprehensive assessment of the association between the plasma sclerostin levels and responsiveness to TNF-inhibitor treatment in RA. Non-responders with active RA would be expected to exhibit abnormal bone turnover with elevated plasma sclerostin levels until disease activity is adequately suppressed. Despite these limitations, this study has provided a novel comparison, analyzing changes in plasma sclerostin levels among women with RA who completed a TNFαI therapy regimen using ETA or ADA. The duration of the study, spanning 15 months, was relatively lengthy, and longer follow-up periods subsequent to the treatment course demonstrated improvements in plasma sclerostin levels among female RA patients. In summary, this study may be considered a promising starting point for controlled long-term trials that further investigate our findings.

## 4. Materials and Methods

### 4.1. Patients and Samples

Fifty female patients with RA (mean ± SD age 47.52 ± 11.91 years) were recruited for this study (Figure 2). All patients fulfilled the 1987 or 2010 American College of Rheumatology (ACR)/European League Against Rheumatism (EULAR) classification criteria for the diagnosis of RA and the Polish National Health Fund (NFZ) eligibility criteria for treatment with TNFαI [38,39]. Patients were excluded if they met any of the following exclusion criteria: age. < 18 years; previous use of biological agents; pregnancy and breastfeeding; acute or recent infectious disease; concomitant diseases affecting bone metabolism; fractures; history of cardiac, renal, psychiatric, endocrine, metabolic, or hepatic disease, including of malignancy; chronic alcoholism. None of the enrolled patients smoked cigarettes or received bisphosphonates or hormone replacement therapy, which could have had some impact on bone turnover. Anti-TNF treatment was administered over a 15-month period following the recommended doses for RA—40 mg every 2 weeks as a subcutaneous (SC) injection for adalimumab; 50 mg every week as an SC injection for etanercept; 400 mg at weeks 0, 2, and 4, and then 200 mg every 2 weeks as an SC injection for certolizumab pegol; and 50 mg once a month as an SC injection for golimumab. Patients were also continuing current antirheumatic therapy, including methotrexate (25 mg/week) and prednisone (≤7.5 mg/day for up to 6 months maximum). All patients were given folic acid at a dose of 5 mg/day. The use of vitamin D (800–1000 IU/day) and calcium (1 g/day) supplements was permitted. Concomitant medications remained unchanged throughout the study duration.

The control group consisted of 26 healthy female volunteers from the Medical University of Silesia in Katowice, Poland. The exclusion criteria for the healthy subjects were hospitalization or surgery within the 3 years prior to the study as well as pharmacological treatment with glucocorticoids or drugs known to affect bone metabolism. None of the volunteers had experience of alcohol addiction. Additionally, subjects were selected following the acquisition of their medical history and laboratory screening. All morphological and biochemical parameters assessed in healthy individuals had to be within the reference ranges. Baseline demographic, clinical and laboratory data of control subjects and patients with RA are presented in Table 6.

All healthy volunteers and RA patients provided written informed consent in accordance with the Helsinki Declaration. The Bioethics Committee of the Medical University of Silesia in Katowice accepted the study protocol (KNW/0022/KB/56/I/12/13).

### 4.2. Clinical and Laboratory Assessments

Routine laboratory screening tests were conducted prior to the administration of the first dose of TNFαI and subsequently at 3, 9 and 15 months post-therapy. The DAS28-ESR and levels of inflammatory markers, such as ESR and CRP, were used to objectively monitor disease activity and patients’ response to 15-month TNFαI treatment. The DAS28-ESR score was calculated from four components: the TJC, SJC, ESR and VAS scores of the patients’ global health. RA female patients who did not experience an adequate response to TNFαI treatment were excluded from the study. According to the principles of the Polish National Health Fund Therapeutic Programs (B.33 or B.45), an adequate treatment response was defined as a reduction in DAS28 greater than 1.2 after the first three months of anti-TNF-α therapy, followed by a further reduction in DAS28 by 1.2 recorded during subsequent medical investigations performed at 9 and 15 months following the administration of the first dose of TNF-α blocker. ESR and CRP were assayed with routinely used methods, which were previously described in our earlier investigations [13,40].

Fasting morning venous blood samples were collected into heparin-treated tubes (measurement of plasma SOST), and aliquots of blood plasma samples were separated within 60 min and stored at −80 °C until further analysis.

#### Immunoassay of Sclerostin

The levels of plasma sclerostin were determined in plasma samples collected at the baseline and 15 months after starting TNFαI therapy. Sclerostin levels were measured using a highly sensitive specific immunoassay from TECOmedical Group (Sissach, Switzerland), in accordance with the instructions provided by the manufacturer. The minimum detection limit of the assay was 0.006 ng/mL. All plasma samples were determined in one day; hence, the inter-assay coefficient of variation (CV) for SOST measurements was insignificant. The intra-assay CV was 4.8%.

### 4.3. Statistical Analysis

The obtained results were processed via the Statistica 13.3 software (TIBCO Software Inc., Palo Alto, CA, USA). The normality of distributions was checked using the Shapiro–Wilk test. Continuous variables with normal distribution were presented as the mean ± SD; non-normal variables were reported as the median and interquartile range (25th–75th percentile). The assumption of the homogeneity of variances was tested using Levene’s test. Comparisons between parameters in women with RA and controls were performed using Student’s *t*-test or the Mann–Whitney U test. Student’s *t*-test or the Wilcoxon signed rank test were used to assess changes in parameters within paired samples of each RA patient. Spearman’s test was used for correlation analysis. Multiple regression analysis of the data was also performed. A *p*-value was considered significant if it was <0.05.

## 5. Conclusions

In summary, we report that long-term anti-TNF-α therapy not only reduces systemic inflammation, but also appears to have a positive effect on bone turnover with a significant reduction in bone metabolism marker SOST, which may reveal an important mechanism for preventing future skeletal damage in patients with RA. The changes in circulating SOST levels, which correlate with the decline in disease activity, suggest that this marker may be valuable in monitoring the effectiveness of TNFαI therapy in patients with RA. However, further studies are needed to definitively verify our results, given the relatively limited number of participants in all studied groups.

## Figures and Tables

**Table 1:** Demographic, clinical and laboratory characteristics of RA patients before the start of TNFαI treatment and at the 15 month of treatment.

Parameter	Woman with RA Which Responded to TNFαI Therapy (*n* = 31)	
	Baseline (T_0_)	15 Months after TNFαI Therapy (T_3_)
Age (years)	45.87 ± 12.28	
Premenopausal females, *n* (%)	17 (54.84)	
Postmenopausal females, *n* (%)	14 (45.16)	
Disease duration (years)	5 (3–11)	
Growth (cm)	163.77 ± 6.63	
Weight (kg)	65.89 ± 14.60	
BMI (kg/m^2^)	24.62 ± 5.65	
IgM-RF (+), *n* (%)	28 (90.32)	
Anti-CCP (+), *n* (%)	26 (83.87)	
ESR (mm/h)	17.0 (10.0–34.0)	13.0 (8.0–18.0) ^a^
CRP (mg/L)	6.3 (3.08–14.0)	4.0 (1.5–5.1) ^a^
Calcium ^C^ (mmol/L)	2.30 ± 0.11	2.31 ± 0.11
Phosphorus (mmol/L)	1.36 ± 0.20	1.37 ± 0.21
ALP (U/L)	168.5 (152.5–202)	165.5 (149.5–192)
SJC28, *n*	7 (5–10)	0 (0–0) ^a^
TJC28, *n*	12 (9–16)	0 (0–0) ^a^
VAS, (0–100 mm)	80 (80–80)	15 (5–20) ^a^
DAS28-ESR	5.78 (5.51–6.24)	2.19 (1.75–2.51) ^a^
Bone mineral density (BMD)		
Lumbar L2-L4 BMD (g/cm^2^)	0.89 (0.73–1.00)	0.92 (0.79–1.03)
Neck femur BMD (g/cm^2^)	0.83 (0.69–0.78)	0.85 (0.77–0.85)
T-score		
Lumbar L2-L4 T-score	−2.05 (−2.93–0.32)	−1.70 (−2.75–−0.65)
Neck femur T-score	−0.30 (−1.30–0.40)	−0.30 (−1.80–0.50)
Z-score		
Lumbar L2-L4 Z-score	−1.43 (−2.38–0.23)	−1.15 (−2.00–0.15)
Neck femur Z-score	−0.10 (−0.90–0.10)	0.00 (−0.70–0.10)
Patients which responded to TNFαI therapy, *n* (%)		
ETA (Enbrel^®^)	16 (51.62)	
ADA (Humira^®^)	13 (41.93)	
CZP (Cimzia^®^)	2 (6.45)	

^a^ Significant differences compared with T_0_. *p* < 0.05. ADA, adalimumab; ALP, alkaline phosphatase; BMD, bone mineral density; ^C^ albumin-corrected calcium; CRP, C-reactive protein; CZP, certolizumab pegol; DAS28-ESR, 28-joint disease activity score based on erythrocyte sedimentation rate; ETA, etanercept; ESR, erythrocyte sedimentation rate; RA, rheumatoid arthritis; SJC28, swollen joint count out of 28 joints; TJC28, tender joint count out of 28 joints; TNFαI, tumor necrosis factor α inhibitors; VAS, visual analogue scale.

**Table 2:** Changes in clinical and laboratory parameters during 15-month TNFαI treatment.

	Woman with RA Which Responded to TNFαI Therapy (*n* = 31)			
Parameter	Time of TNFαI Therapy (ADA or ETA or CZP)			
	Baseline (T_0_)	3 mths. (T_1_)	9 mths. (T_2_)	15 mths. (T_3_)
The inflammatory variables				
ESR (mm/h)	17.0 (10.0–34.0)	14.0 (9.0–23.0)	13.0 (9.0–18.0) ^a^	13.0 (8.0–18.0) ^a^
CRP (mg/L)	6.3 (3.08–14.0)	4.0 (2.0–9.0)	4.0 (2.0–4.3) ^a^	4.0 (1.5–5.1) ^a^
The clinical variables				
SJC28, *n*	7 (5–10)	2 (0–3) ^a,c^	0 (0–0) ^a,b^	0 (0–0) ^a,b^
TJC28, *n*	12 (9–16)	4 (2–7) ^a,c^	1 (0–2) ^a,b^	0 (0–0) ^a,b,c^
VAS, (0–100 mm)	80 (80–80)	40 (30–50) ^a,c^	20 (10–30) ^a,b^	15 (5–20) ^a,b^
DAS28-ESR	5.78 (5.51–6.24)	3.92 (3.08–4.42) ^a,c^	2.75 (2.24–3.13) ^a,b^	2.19 (1.75–2.51) ^a,b,c^
Disease activity, *n* (%)				
High (>5.1)	31 (100)	2 (6.45)	0 (0)	0 (0)
Moderate (>3.2 and ≤5.1)	0 (0)	20 (64.52)	3 (9.68)	0 (0)
Low (≤3.2 and >2.6)	0 (0)	4 (12.91)	14 (45.16)	5 (16.13)
Remission (≤2.6)	0 (0)	5 (16.13)	14 (45.16)	26 (83.87)

*p* < 0.0083 (Bonferroni correction). ^a^ Significant differences compared with T_0_; ^b^ significant differences compared with T_1_; ^c^ significant differences compared with T_2_. ADA, adalimumab; CRP, C-reactive protein; CZP, certolizumab pegol; DAS28-ESR, 28-joint disease activity score based on erythrocyte sedimentation rate; ETA, etanercept; ESR, erythrocyte sedimentation rate; RA, rheumatoid arthritis; SJC28, swollen joint count out of 28 joints; TJC28, tender joint count out of 28 joints; TNFαI, tumor necrosis factor α inhibitors; VAS, visual analogue scale.

**Table 3:** Plasma sclerostin levels in RA patients before the start of TNFαI treatment and at the 15 month of treatment, according to stage of menopause.

Parameter/ Stage of Menopause	SOST [ng/mL]		
	Baseline (T_0_)	15 Months after TNFαI Therapy (T_3_)	*p*-Value
Premenopausal Patients (*n* = 17)	0.72 ± 0.24	0.61 ± 0.26	**0.015**
Postmenopausal Patients (*n* = 14)	0.88 ± 0.22	0.78 ± 0.20	**0.027**
*p*-Value	0.060	0.061	

Bold values are significant at *p* < 0.05. SOST, sclerostin; TNFαI, tumor necrosis factor α inhibitors.

**Table 4:** Correlations between plasma sclerostin levels and demographic, clinical and laboratory parameters of RA patients before the start of TNFαI treatment and at the 15 month of treatment.

Parameter	Woman with RA Who Responded to TNFαI Therapy (*n* = 31)			
	SOST (ng/mL)			
	Baseline (T_0_)		15 Months after TNFαI Therapy (T_3_)	
	r	*p*	r	*p*
Age (years)	0.490	**0.005**		
Disease duration (years)	0.342	0.059	0.426	**0.017**
ESR (mm/h)	0.428	**0.016**	0.220	0.235
CRP (mg/L)	0.249	0.177	0.078	0.678
ALP (U/L)	0.013	0.953	−0.192	0.370
SJC28, *n*	−0.235	0.203	0.085	0.648
TJC28, *n*	−0.095	0.612	0.349	0.055
VAS, (0–100 mm)	0.115	0.538	0.222	0.230
DAS28-ESR	0.417	**0.020**	0.468	**0.008**

Bold values are significant at *p* < 0.05. ALP, alkaline phosphatase; CRP, C-reactive protein; DAS28-ESR, 28-joint disease activity score based on erythrocyte sedimentation rate; ESR, erythrocyte sedimentation rate; RA, rheumatoid arthritis; SJC28, swollen joint count out of 28 joints; SOST, sclerostin; TJC28, tender joint count out of 28 joints; TNFαI, tumor necrosis factor α inhibitors; VAS, visual analogue scale.

**Table 5:** Multivariate regression analysis for predictors of baseline plasma SOST levels in RA patients.

Parameter	SOST in RA Patients (*n* = 31) at Baseline (T_0_)	
	β	*p*-Value
Age [years]	0.008	**0.028**
DAS28-ESR	0.128	0.134

Bold value is significant at *p* < 0.05. SOST, sclerostin; DAS28-ESR, 28 joint disease activity score based on erythrocyte sedimentation rate; ESR, erythrocyte sedimentation rate; RA, rheumatoid arthritis.

**Table 6:** Baseline characteristics of control subjects and RA patients before TNFαI treatment.

Parameter	Healthy Controls	RA Patients at Baseline (T_0_)	*p*-Value
	*n* = 26	*n* = 31	
Age (years)	46.12 ± 10.91	45.87 ± 12.28	0.938
Premenopausal females, *n* (%)	16 (61.54)	17 (54.84)	–
Postmenopausal females, *n* (%)	10 (38.46)	14 (45.16)	–
Disease duration (years)	–	5 (3–11)	–
Growth (cm)	166.64 ± 6.23	163.77 ± 6.63	0.386
Weight (kg)	64.0 (62.0–67.0)	64.0 (57.0–70.0)	0.797
BMI (kg/m^2^)	23.65 (22.15–24.84)	24.09 (21.01–25.91)	0.382
IgM-RF (+), *n* (%)	0 (0)	28 (90.32)	–
Anti-CCP (+), *n* (%)	0 (0)	26 (83.87)	–
RBCs (10^6^/µL)	4.28 ± 0.23	4.22 ± 0.24	0.380
Hb (g/dL)	13.08 ± 0.97	12.89 ± 1.20	0.562
Ht (%)	38.68 ± 3.10	38.31 ± 3.47	0.703
Platelet (10^3^/µL)	263.88 ± 49.43	258.20 ± 69.70	0.748
WBCs (10^3^/μL)	8.16 ± 1.63	7.79 ± 1.79	0.461
ESR (mm/h)	9.0 (8.0–12.0)	17.0 (10.0–34.0)	**<0.001**
CRP (mg/L)	0.61 (0.40–2.81)	6.3 (3.08–14.0)	**<0.001**
Calcium ^C^ (mmol/L)	2.28 ± 0.09	2.30 ± 0.11	0.324
Phosphorus (mmol/L)	1.34 ± 0.25	1.36 ± 0.20	0.698
ALP (U/L)	161.0 (142.0–172.0)	168.5 (152.5–202.0)	0.066
ALT (U/L)	20.50 (13.0–26.0)	21.50 (13.50–32.0)	0.405
AST (U/L)	19.0 (17.0–24.0)	20.50 (18.0–26.50)	0.209
Creatinine (mg/dL)	0.86 (0.81–0.92)	0.70 (0.58–0.80)	**<0.001**
SJC28, *n*	–	7 (5–10)	–
TJC28, *n*	–	12 (9–16)	–
VAS, (0–100 mm)	–	80 (80–80)	–
DAS28-ESR	–	5.78 (5.51–6.24)	–

Bold values are significant at *p* < 0.05. anti-CCP, anti-cyclic citrullinated peptide antibody; ALP, alkaline phosphatase; ALT, alanine transaminase; AST, aspartate transaminase; ^C^ albumin-corrected calcium; BMI, body mass index; CRP, C-reactive protein; DAS28-ESR, 28 joint disease activity score based on erythrocyte sedimentation rate; ESR, erythrocyte sedimentation rate; Hb, hemoglobin; Ht, hematocrit; RA, rheumatoid arthritis; RBCs, red blood cells; RF, rheumatoid factor; SJC28, swollen joint count out of 28 joints; TJC28, tender joint count out of 28 joints; TNFα, tumor necrosis factor α inhibitor; WBCs, white blood cells; VAS, visual analogue scale.

## Data Availability

Data are contained within the article.
